# PATH Trial for Examining Yoga as a Strategy to Improve Remote-Based Weight Loss in Adults: Protocol for a Randomized Controlled Trial

**DOI:** 10.2196/78973

**Published:** 2026-02-25

**Authors:** Selene Y Tobin, Sally Sherman, Beth Bock, Shira Dunsiger, J Graham Thomas, Stephanie P Goldstein, Jessica Unick

**Affiliations:** 1 Weight Control and Diabetes Research Center, The Miriam Hospital, 196 Richmond Street, Providence, RI, 02903, United States, 1 401 793 8966; 2 Department of Psychiatry and Human Behavior, Brown University, Providence, RI, United States; 3 Department of Health and Kinesiology, California Polytechnic University, San Luis Obispo, CA, United States; 4 Department of Health and Human Development, Physical Activity Research Center, University of Pittsburgh, Pittsburgh, PA, United States; 5 Department of Behavioral and Social Sciences, Brown University, Providence, RI, United States

**Keywords:** internet-delivered weight loss, long-term weight loss, yoga, randomized controlled trial, obesity, overweight

## Abstract

**Background:**

Over 70% of US adults are overweight or have obesity. Internet-delivered weight loss (IDWL) treatment overcomes many common barriers to in-person weight loss (WL) programs; yet, IDWLs underperform compared to in-person treatment. Yoga is a novel intervention that targets physical and psychological health and can be delivered virtually, increasing translation potential. Yoga has been understudied as an intervention to address barriers to WL despite favorable effects in other health disciplines. Preliminary studies suggest yoga to improve self-regulation and reduce lapses from dietary recommendations, 2 factors integral to long-term WL success.

**Objective:**

The aim of the study is to conduct the first fully powered randomized controlled trial to test the added effect of yoga to IDWL treatment on weight (primary outcome), dietary lapses, potential lapse triggers, and self-regulation mediator variables (secondary outcomes).

**Methods:**

Adults that are overweight or have obesity will be recruited to participate in a 12-month IDWL program. Following a 3-month assessment, eligible participants will be randomized to (1) a yoga intervention, including 14 weeks of group-based, virtual Iyengar yoga delivered twice weekly, followed by 22 weeks of yoga delivered once per week, and then 6 months of no contact, or (2) a contact-matched wellness comparison condition. Assessments will take place at baseline, 3 months, 6 months, 12 months, and 18 months. Several questionnaires, ecological momentary assessments, and objectively measured weight and accelerometry data will be collected. This trial is registered on ClinicalTrials.gov (NCT06166225).

**Results:**

This randomized trial was funded with an original project period of July 2023-June 2028. Enrollment began in February 2024 and is ongoing with the expectation of closing enrollment in June 2026.

**Conclusions:**

This trial will examine the combined effect of yoga and WL treatment among individuals with overweight or obesity and will be the first fully powered trial to assess the combination of virtually delivered yoga and WL beyond 6 months. Findings will inform whether yoga could be considered a valuable intervention component for improving IDWL treatment.

## Introduction

Over 70% of US adults are overweight or have obesity [1]. Internet-delivered weight loss (IDWL) treatment overcomes many common barriers to in-person weight loss (WL) programs (eg, travel, childcare, and geographical constraints), and emerging evidence indicates that online delivery of wellness programs is effective and acceptable [2-5]. Nonetheless, IDWL often underperforms when compared to in-person treatment, and weight regain following WL is still highly prevalent [6,7]. Thus, novel approaches for improving long-term IDWL are needed.

Yoga is an ancient practice that encompasses physical and mental elements that promote overall well-being, flexibility, strength, and mental clarity [8]. Yoga is often most recognized for its physical postures, which are associated with movement and physical activity (PA), but its broader benefits extend to emotional and psychological health. Interdisciplinary findings demonstrate the downstream effects of yoga to improve aspects of all 3 dimensions of self-regulation. This includes having a favorable impact on mindfulness and distress tolerance (emotion regulation), inhibitory control (cognitive regulation), self-efficacy, and self-compassion (self-related processes) [9-29]. These effects are of particular interest, as prior obesity medicine research indicates that individuals successful at long-term WL have high self-regulatory capacity and exhibit more positive self-related processing [30-36]. Therefore, yoga may offer a novel pathway to cultivate these weight-related behavioral skills. Yoga may also reduce dietary lapses and improve WL via improvements in psychological factors that are associated with or trigger a lapse. For example, yoga has been shown to improve affect (ie, improved positive affect and reduced negative affect) [37,38], and prior research shows that more negative affect is associated with increased frequency of dietary lapses [31,39,40]. Together, these findings suggest that yoga targets multiple self-regulatory and affective processes, which can exert a synergistic effect on eating behavior and body weight. Thus, the rationale for using yoga to reduce dietary lapses and improve long-term WL in IDWL treatment is strong.

To date, yoga has largely been overlooked within the context of WL treatment, despite positive effects observed in other health disciplines (eg, cardiovascular disease risk, pain, depression, and smoking cessation) [41-44]. While other disciplines focus on the psychological benefits of yoga, the weight control field has traditionally viewed yoga as a *mode* of exercise (characterized by low energy expenditure and classified as light PA that may not elicit a sufficient caloric deficit to contribute to WL), thereby overlooking the cognitive and emotional pathways through which yoga may enhance WL. Although cross-sectional evidence suggests strong associations between yoga and healthy dietary choices, mindful eating, and emotional eating [25,45-47], studies investigating the inclusion of yoga within WL treatment are limited. A 2021 systematic review that explored relationships between yoga participation and reductions in body weight via decreased energy intake or increased PA identified only 2 prior randomized controlled trials (RCTs), which combined yoga with calorie reduction in individuals with overweight or obesity [48]. The first was a 6-month study conducted among 50 individuals with overweight or obesity and compared 2 styles of yoga, restorative hatha and vinyasa. While this study demonstrated yoga participation within a standard behavioral WL intervention to be feasible, no differences in WL were observed between the 2 styles of yoga, as both groups lost 3.4‐3.8 kg [49]. Further, because it did not have a control condition, any conclusion related to the added effect of yoga on WL is limited. The second study was a 12-week trial conducted in 260 individuals diagnosed with metabolic syndrome and was conducted outside of the United States (India), where yoga practice differs from the United States [50]. Results suggested that a greater proportion of participants randomized to the yoga-based intervention recovered from metabolic syndrome (45.4%) as compared to dietary intervention alone (32.3%). However, energy cost was not controlled for in this study, and observed effects may have been due to elevated PA levels.

We recently published findings from our pilot RCT (n=60), wherein 60 female individuals with overweight and obesity were randomized to a 3-month yoga program or a contact-matched control (cooking or nutrition classes) following 3 months of behavioral WL treatment [4,51]. Adherence (attendance to the yoga classes was 75%) and retention (100%) were high. Yoga participants reported fewer lapses from the recommended diet (eg, via less overeating, stress eating, and loss of control when eating) and less difficulty resisting dietary temptations compared to the control group. Post-hoc analyses revealed that yoga was associated with a larger magnitude of 6-month WL and several self-regulatory skills (mindfulness, distress tolerance, and self-compassion) compared to the control, among those who lost more weight in the first 3 months of treatment (≥5%). These results led to the hypothesis that yoga can not only decrease the likelihood of dietary lapse but also reduce many common factors, which could potentially trigger a dietary lapse contributing to longer-term WL success. Additionally, in this pilot trial, we found online delivery of a yoga program to be feasible. Specifically, due to COVID-19, one cohort in this study received yoga classes remotely (live via videoconferencing), and attendance and satisfaction ratings were comparable to in-person yoga classes administered in the previous cohort. These preliminary findings demonstrate initial feasibility and acceptability of including yoga within the context of a WL program and indicate that yoga can reduce dietary lapses and improve self-regulation, 2 factors important for long-term weight control.

The purpose of this study, PATH Weight Loss Trial (Pathway to Achieving Total Health and Weight Loss), is to extend upon this pilot study and address important limitations in the current literature in several ways. First, this will be the first fully powered RCT to assess the added effect of yoga to IDWL treatment on longer-term WL outcomes (primary outcome is 12-month WL [immediately after the intervention], and secondary outcome is 18-month WL [following a 6-month no intervention period]). Investigation of long-term WL outcomes is currently a high priority of obesity medicine, as high rates of weight regain following WL persist. This trial also extends the literature by examining the effect of yoga on dietary lapses and potential lapse triggers (eg, affect, craving, and dietary temptations) to better understand how yoga may influence eating behaviors within the context of WL treatment. It will also examine potential self-regulatory mechanistic pathways through which yoga may impact WL and dietary lapses, which have not yet been explored within WL treatment. Finally, it is also anticipated that results will provide a higher degree of generalizability than standard weight control literature by including male individuals and greater racial or ethnic diversity than previous literature.

## Methods

### Study Setting

The PATH Trial is a randomized clinical trial funded by the National Center for Complementary and Integrative Health (R01AT011868) and is registered on ClinicalTrials.gov (NCT06166225). While the trial is entirely remote, the study will be conducted out of the Weight Control and Diabetes Research Center in Providence, Rhode Island. The Weight Control and Diabetes Research Center is a joint research institution affiliated with Brown University Health and The Warren Alpert Medical School at Brown University.

### Ethical Considerations

All study procedures were developed in accordance with the Declaration of Helsinki ethical principles and approved by the institutional review board at Brown University Health (IRB # 1947629). Participants will be asked to complete informed consent via REDCap (Research Electronic Data Capture; Vanderbilt University) after attending an online study orientation session with a member of the research team. Participants will be eligible for compensation up to US $75 in the form of a gift card for the completion of assessments at 3, 6, 12, and 18 months. The US $75 compensation is calculated in the following manner: participants will receive US $25 for completing the online questionnaires and wearing the PA monitor; they will also be compensated US $0.50 for each ecological momentary assessment (EMA) survey completed (50 surveys per assessment period) and will receive a US $25 bonus for EMA compliance >80%. No compensation will be provided for the baseline assessment visit. The full study protocol, informed consent document, and study results will be published on ClinicalTrials.gov upon study completion. To protect participant confidentiality, all study data collected via our secure website using an active Secure Sockets Layer or Transport Layer Security certificate (HTTPS), or other platforms, will be securely stored on institutionally managed, access-controlled servers protected by firewalls, multifactor authentication, and regular security monitoring. All physical data will be stored securely in locked cabinets, with access strictly limited to authorized and trained study personnel.

### Study Overview

**Figure 1.**
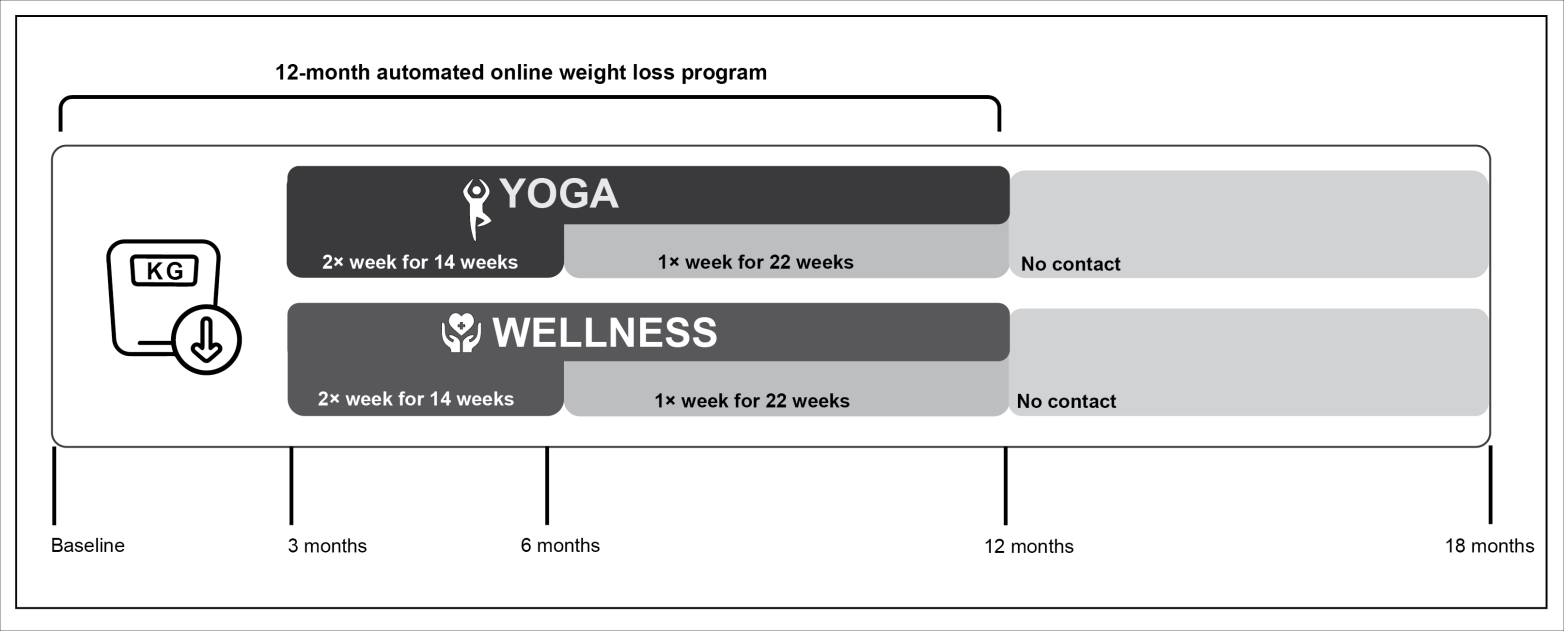
Study design. Participants will complete a 12-month weight loss program. Following 3 months of weight loss, participants will be randomized to either yoga or the wellness comparison condition. Yoga and wellness classes will be held twice weekly for 14 weeks, then reduced to once weekly for 22 weeks, followed by a 6-month period of no contact. Study assessments will take place at baseline, 3 months (immediately following weight loss, prior to intervention randomization), 6 months, 12 months, and 18 months.

This protocol is reported in accordance with the SPIRIT (Standard Protocol Items: Recommendations for Interventional Trials) 2025 guidelines for randomized trials, and any significant modifications to the protocol will require prior approval from the study sponsor. An overview of the study design is shown in Figure 1. A total of 210 participants will enroll in this randomized controlled clinical trial with a parallel-group design and equal allocation. All participants will receive a 12-month IDWL program, and those completing the 3-month assessment visit will be randomized to receive (1) a 9-month yoga program (YOGA; delivered during months 4‐12) or (2) a 9-month contact-matched control “Health & Wellness program” (WELLNESS; delivered during months 4‐12). The choice to delay randomization to YOGA or WELLNESS until after the 3-month assessment, rather than at baseline, was made to give participants time to build foundational WL skills without added burden and to reinforce self-regulatory skills at a time (3 months) when WL-related motivation may begin to dip [7,52]. Additional rationale to delay randomization to YOGA or WELLNESS is provided in the discussion. A full description of all intervention components is detailed below and conforms with the TiDieR (Template for Intervention Description and Replication) checklist for intervention reporting to enhance clarity and replicability.

Yoga and wellness classes will be delivered via videoconferencing software (ie, Health Insurance Portability and Accountability Act [HIPAA]–compliant Zoom; Zoom Video Communications). There will be no intervention during months 13‐18. Randomization will occur at the 3-month assessment (ie, 3 months into the IDWL) in equal numbers based on a permuted block randomization procedure with small, random sized blocks, stratified by biological sex, racial or ethnic minority status, and percent WL achieved during the first 3 months of the WL program (<5% vs ≥5% WL). The randomization scheme was generated by the study statistician using R Studio (version 3.6.0) and uploaded into REDCap directly (to ensure study staff was kept blinded toward the full schematic). Participants will be recruited in cohorts over the study period. Assessments of weight, aerobic PA minutes, dietary lapses, potential lapse triggers (eg, affect, craving, and dietary temptations), and self-regulation mediator variables will be measured at baseline, 3 months (just prior to randomization), 6 months (mid-treatment), 12 months (postintervention), and 18 months (following a 6-month period of no intervention). At each assessment period, data will be collected via a series of REDCap questionnaires, an Actigraph accelerometer, and a 10-day EMA protocol (ie, 5 surveys per day over 10 days with each survey taking 2‐5 minutes to complete).

### Study Aims

The primary aim of the PATH Trial is to compare the treatment effects of IDWL and YOGA versus IDWL and WELLNESS on weight change at 12 months. The primary weight outcome is absolute weight change from baseline to 12 months. It is hypothesized that WL will be greater in those randomized to the YOGA when compared to WELLNESS.

Secondary aims of this study are (1) to compare YOGA and WELLNESS at 12 months on dietary lapses and potential lapse triggers as assessed via EMA and (2) to examine self-regulation mediators (eg, mindfulness, self-compassion, distress tolerance, self-efficacy, and inhibitory control) of the treatment effect on primary (ie, weight change) and secondary outcomes (ie, dietary lapses) at 12 months.

Exploratory aims are to examine the long-term (18 months) effects of yoga on primary and secondary outcomes, to explore potential moderators (3-month WL, race or ethnicity, biological sex, and BMI) of the treatment effect on weight change, and to explore the effect of yoga on participation in aerobic exercise.

### Study Participants, Eligibility Criteria, and Recruitment Procedures

A total of 210 WL-seeking male and female individuals will be recruited for this study. Specifically, participants will be between the ages of 18 and 65 years and have a BMI of 25‐40 kg/m^2^, regardless of race or ethnicity. Participants must own a smartphone (for EMA surveys) and have daily, home internet access that allows for videoconferencing. Exclusion criteria will include currently pregnant, planning to become pregnant within the next 18 months, or pregnant within the past 6 months; current or recent enrollment (<2 years) in a WL program; current or recent yoga, meditation, or mindfulness practice, either at home or in a studio (participants who reported ≥12 sessions within the past 2 years will be followed up with to confirm eligibility); presence of any medical condition for which yoga, exercise, WL, or dietary restriction is contraindicated (Physical Activity Readiness Questionnaire); recent WL (≥4.5 kg within the past 6 months); currently taking WL medications or history of bariatric surgery; inability to walk 2 blocks without stopping; failure to complete all baseline assessment procedures; and baseline EMA compliance <80% (to be used as an indicator of behavioral adherence). To ensure safety, individuals with a history of heart disease, diabetes, cancer, or major orthopedic conditions will be required to provide physician consent prior to enrolling.

Participants will be recruited through online advertisements via social media outlets. Participants will be asked to respond to advertisements by completing an online screener linked to the advertisement. Participants will be recruited in 5 cohorts with a targeted cohort size of 35‐60 participants. Individuals who meet all inclusion criteria and are eligible to participate in the study will be invited to attend a study orientation (delivered via Zoom), in which the study will be explained in greater detail. Individuals who attend orientation and remain interested in participating in the study will be emailed a link to complete informed consent via REDCap [53]. Data will be collected and managed using the REDCap tools hosted at Brown University Health’s Department of Information Services. REDCap is a secure, web-based application designed to support data capture for research studies. Once consent is obtained, participants will begin the baseline assessment, which consists of study questionnaires, an EMA protocol, and wearing an accelerometer. Further, as part of the assessment, participants are required to attend a one-on-one behavioral interview via videoconference with a member of the research staff to ensure that the participant is willing to commit to all study procedures prior to beginning the WL program.

### Internet-Based WL Program

All participants will receive a 12-month, fully automated IDWL program, which has been used in previous trials [7,54,55]. Prior to the start of the program, participants will be asked to watch a brief video, which will provide them with the basics for navigating the study website. The goal of the program is to induce a 1‐2 lb (~0.5‐1 kg) WL per week. To assist in achieving this goal, individuals will be given an initial daily calorie intake goal of 1200‐1800 kcal per day depending upon starting weight. Participants will also be taught to increase or decrease calorie intake as necessary based on WL progress and goals. There will be also a moderate-intensity aerobic exercise (eg, walking, cycling, and elliptical) goal, which progresses to 150 minutes per week. Participants will be instructed to self-monitor body weight, energy intake, and PA daily over the 12-month WL period, as self-monitoring in a behavioral WL program has been shown to be associated with weight change [56]. They will be provided with a smart scale, which will allow for wireless [57] transmission of weight data to the study website. Participants will be given the option to track their calories and exercise minutes in the Fitbit mobile app, in which case data will automatically transfer to the study website through an application programming interface built specifically for this study. However, if they use a different tracking method, they will be asked to manually input their daily calorie intake and exercise minute totals on the study website. Computer-generated, personalized feedback will be provided using these self-report data. Participants will also be instructed to view 10‐ to 15-minute multimedia lessons, which are modeled after the Look AHEAD Trial [57]. Example lesson topics including strategies for increasing PA, eating out at restaurants, and problem-solving. Video lessons and automated feedback are provided weekly during the first 3 months of the IDWL program and monthly during months 4‐12. Participants also have access to healthy recipes and tip sheets (eg, meal planning strategies, eating healthy on a budget, and exercising safely) via the study website. During months 4‐12, automated text messages, which are informational and motivational in nature, will also be sent to participants twice per month and will differ between yoga and wellness conditions.

### YOGA Intervention

#### Overview

Upon completion of the 3-month assessment visit, participants randomized to YOGA will receive a 9-month fully remote, Iyengar yoga intervention, which will include live, virtual classes (delivered via Zoom with real-time feedback provided) and self-guided, home-based practice using prerecorded videos. All live and prerecorded group classes will be led by a certified Iyengar yoga teacher, who will be trained using a standardized manual of procedure developed by the research team in collaboration with the yoga instructors. Iyengar yoga is a form of hatha yoga, which incorporates breathing, postural, and meditation practices and focuses on the precision in movement, maintaining proper alignment, and attention to subtle aspects of posture and breath. Iyengar yoga was chosen as the style of yoga for this study due to the precision in cueing, the use of props to modify postures for participants based on personal needs, and to minimize the risk of injury. Additionally, Iyengar is believed to have a lower energy expenditure than other more vigorous styles of yoga (eg, vinyasa or power yoga). Furthermore, the yoga curriculum was designed to ensure that it was inclusive to participants of any comfort level with yoga or existing assumptions about the practice. For example, there was no chanting of “OM” in the classes, and if yoga teachers used Sanskrit terms, they would always translate the names of the poses in English.

#### Introduction to Yoga Content

All participants will be mailed a yoga mat, strap, and 2 yoga blocks to facilitate home-based practice. At the start of the yoga program, participants will be required to complete an “Introduction to Yoga” curriculum (2 classes per week for 2 weeks) prior to engaging in the larger group classes. Each of these 60-minute introductory sessions will provide guidance on how to set up the home environment to foster a regular yoga practice, teach the proper form and alignment for foundational yoga poses, familiarize participants with the format of a yoga practice, and allow participants the opportunity to ask questions. Participants will practice yoga in front of their web camera so that instructors can provide individualized feedback to ensure that poses are performed properly and safely.

#### Live, Virtual Classes

Following the completion of the “Introduction to Yoga” curriculum, participants will advance to the larger group, 60-minute live classes. These classes will be delivered via Zoom for 36 weeks to guide participants through the yoga practice. Modifications will be provided for each pose to ensure that the level of difficulty is suitable for all individuals. Walls and chairs will be used for stability if needed. Group classes will be held multiple times per week. Participants will be instructed to attend at least 2 classes per week for the first 3 months (following the 2-week introduction, for a total of 14 weeks) and at least 1 class per week until the 12-month study assessment (22 weeks total). To increase translation potential, yoga classes will be open groups, meaning that participants may attend classes on days and times that best fit their needs, and participants from multiple cohorts will attend sessions together.

#### Self-Guided Yoga Practice

In addition to participating in the live, virtual classes, participants will also be given a self-guided yoga prescription to assist in the development of their personal practice. To facilitate self-guided practice, participants will be instructed to use video recordings from the study website, which will offer classes from the study’s Iyengar teachers of varying durations (eg, 15, 30, 45, and 60 minutes). The first 3 months will focus more heavily on live yoga instruction, and the last 6 months will be designed to foster a more autonomous practice. The self-guided prescription is based upon total duration (ie, minutes per week) rather than frequency (ie, days per week) to facilitate greater retention and adherence by allowing individuals the choice to practice yoga less frequently for a longer duration (eg, 1 time per week for 60 minutes) or more frequently for shorter durations (eg, 3 times per week; one, 30-minute class and two, 15-minute classes). Progression toward a total dose (ie, combination of live classes and self-guided practice) of 180 minutes per week of yoga practice is the intervention target for both the first 3 months and the final 6 months of the program.

#### Verbal Cues for Life Application (Yamas or Niyamas)

Asanas (ie, the physical practice of yoga poses) taught within classes will be infused with verbal teachings of yamas or niyamas (ie, social disciplines to guide relationships with oneself and others) to help cue participants into how yogic philosophies can overflow into everyday life (ie, how to apply skills practiced within class to personal experiences or struggles encountered outside of class). To ensure translatability of the yoga intervention, verbal cues will not explicitly discuss application to WL but will be broad enough so that participants can apply these principles to their weight control practices. Table 1 describes the 5 yamas and 5 niyamas, which simultaneously target several of the self-regulatory mechanisms examined within this study (eg, distress tolerance, mindfulness, and self-compassion). It is also important to note that many weight control behaviors can relate to several yamas and niyamas at once. For instance, choosing to eat and drink in moderation (vs overeating) so that one has more energy to engage in activities that one values can involve brahmacharya (moderation), aparigraha (nongreed), and satya (truth).

**Table 1.** Yamas and niyamas used in yoga.

Practice		Description
Yamas		
	Ahimsa	Nonviolence; making sure not to harm oneself or others; not thinking negative thoughts; kindness and consideration.
	Satya	Truthfulness; being truthful in one’s thoughts, feelings, speech, and actions; being honest with oneself and others.
	Asteya	Nonstealing; not coveting; not being jealous; letting go of cravings. The need to “steal” often arises from a lack of faith in oneself.
	Brahmacharya	Moderation and channeling emotions; having moderation in our actions; conserving energy (slowing down); showing restraint.
	Aparigrapha	Nongreed; nonpossessiveness; greedlessness; simplicity; fulfilling needs rather than wants.
Niyamas		
	Saucha	Purification; cleanliness, internally and externally; purifying the body, mind, and surroundings.
	Santosha	Contentment; detaching from desires to cultivate inner peace.
	Tapas	Self-discipline; using self-discipline to burn out impurities of mind and body.
	Syadhyaha	Self-reflection; the practice of introspection; self-study.
	Ishvara Prandihana	Surrender; surrendering to a higher power.

#### Additional Yoga-Related Website Components

Instructions on how to properly and safely perform each yoga pose will be available on the study website, along with prerecorded videos for self-guided practice. To assist individuals with making the connection between weight control practices and what they are learning “on the yoga mat,” a brief statement applying a yama or niyama to a WL behavior will be added to the text messages that are sent out twice per month as a part of the IDWL program. Participants will self-monitor their yoga practice and log the daily number of yoga practice minutes on the study website.

#### Recommended Yoga Practice Following the 9-Month Yoga Program

The yoga prescription is designed to facilitate an autonomous practice, which could be continued after the intervention. Upon completion of the 9-month yoga intervention, participants will be encouraged to continue their personal yoga practice of at least 180 minutes per week with the tools taught to them during the intervention. Although no intervention will be provided during months 13‐18, participants will have continued access to the prerecorded videos, and participants will be asked to complete monthly surveys reporting on their yoga practice.

### Contact Control: Health and Wellness Program

Upon completion of the 3-month assessment visit, participants randomized to WELLNESS will receive a 9-month fully remote, health and wellness program. Similar to YOGA, wellness classes will be approximately 60 minutes in duration and held multiple times per week. Participants will be instructed to attend 2 classes per week for the first 14 weeks and 1 class per week until the 12-month study assessment (22 weeks). Classes will be taught by individuals with a bachelor’s degree or higher in a wellness-related field (eg, nutrition, exercise science, health promotion, and psychology). Classes will consist of lectures, handouts, videos, and discussions addressing a variety of health and lifestyle topics, which include immune health, sleep recommendations, health screenings, healthy aging, vitamins and minerals, and humor and health. WL will not be discussed within these sessions, and participants will be informed that wellness classes are designed to function independently of the IDWL program. Overall, the WELLNESS condition provides a treatment program, which serves as a contact-matched control for the yoga arm.

### Fidelity

All live intervention sessions (YOGA and WELLNESS) will be audio-recorded using a HIPAA-compliant version of Zoom. Recordings will be downloaded weekly and stored on a secure server. On a quarterly basis, 15% of all completed class recordings will be assessed for treatment fidelity via checklists developed by the research team for the YOGA and WELLNESS classes. These checklists include items such as class duration, whether the instructor taught the class as intended, and whether there was an opportunity for participants to ask questions. Fidelity will be assessed by a member of the research team who does not interact with participants. If at any time fidelity is <80%, teachers will be retrained and instructed on how to implement any necessary changes.

### Outcome Measures

#### Overview

Assessments of weight, aerobic exercise minutes, dietary lapses, potential lapse triggers, and self-regulation mediator variables will be measured at baseline, 3 months (just prior to randomization), 6 months (mid-treatment), 12 months (postintervention), and 18 months (following a 6-month period of no intervention). Both questionnaires and EMA will be used. Research staff members will not be blinded to group assignment, and efforts will be made to collect data from all enrolled participants at the time of each assessment, including those who may no longer be attending intervention classes. Brief descriptions of study measures are provided below.

#### Anthropometric Measures

Weight change throughout the intervention period will be objectively assessed using Wi-Fi and Bluetooth-enabled smart scales (Withings Body), which will be mailed to study participants upon enrollment. At each assessment period, participants will be instructed to weigh first thing in the morning before eating or drinking, without clothing, or in light clothing. They will be asked to weigh 3 consecutive times, and if the maximum difference between any 2 weight measurements is ≤0.5 kg, the mean of the 3 weights will be used in the analysis. If the difference between any 2 weights is >0.5 kg, the weight that is farthest from the median will be dropped, and the mean of the remaining 2 weights will be used. Height will be self-reported, which has high concordance with stadiometer-assessed height measurements [58].

#### Ecological Momentary Assessment

##### Overview

Participants will complete brief surveys for 10 days at each assessment period. All entries will be made in response to 5 semirandom prompts delivered throughout the day via text message. Upon receiving a text message with a one-time link to the survey, participants will have 45 minutes to answer the questions. Each survey begins with the following message: “When completing this survey, please read the questions carefully as some may ask about how you feel ‘right now,’ others may ask what you may have experienced ‘since the last survey,’ and some questions may allow you to ‘select all response options that apply’ versus just one option. Please be as honest and accurate as possible. Your genuine input will help us gain valuable insights into your daily experiences.” Each survey will take 2‐5 minutes to complete.

##### Dietary Lapses and Potential Lapse Triggers

At each survey, participants will be asked if they ate since the last EMA prompt. If eating was endorsed, a series of questions related to dietary lapse will be asked in addition to questions about potential triggers for lapses. These questions are included in Table 2. When eating is not reported, participants will receive a set of supplemental questions, which do not directly address study aims, to maintain consistency in survey structure and reduce response bias. This approach is consistent with previous work studying dietary lapse [39].

**Table 2.** Summary of ecological momentary assessment variables to be collected.

Variable	Measure	Response options
Dietary lapse		
Dietary lapse (type or total)^a^	Since your last survey (even if it was yesterday), at how many meals or snacks did any of the following occur? (a) Ate past the point of feeling full. (b) Ate a bigger portion than you meant to. (c) Had unplanned eating (ie, ate or drank when you do not usually eat or drink and were not making up for a missed meal). (d) Ate a food that you were intending to avoid or reduce. (e) Went over a calorie goal that you had in mind for yourself.	Number of lapse events
Dietary lapse (frequency)^b^	On the previous question, you indicated that since the last prompt, at least one of the following occurred at XX meals or snacks since the last survey (number from above question will be piped in). Now we want to know at how many meals or snacks did each of the following occur: (1) Ate past the point of feeling full. (2) Ate a bigger portion than you meant to. (3) Had unplanned eating (ie, ate or drank when you do not usually eat or drink and were not making up for a missed meal). (4) Ate a food that you were intending to avoid or reduce. (5) Went over a calorie goal that you had in mind for yourself.	Number of each type of lapse
Potential dietary lapse triggers		
Dietary temptation (type)^c^	Since the last survey (even if it was yesterday), have you experienced any of the following temptations to eat or drink, regardless of whether or not you actually acted on the temptation (check all that apply)?	(a) I was tempted to overeat, (b) I had a sudden urge to eat a tempting food or drink that I have been trying to reduce or avoid, (c) I was exposed to a tempting food or drink, (d) none of the above
Dietary temptation (details)^d^	If a, b, or c are selected from the question above, the following questions will be asked: (1) thinking about the most recent temptation you had: what was the intensity of the temptation? (2) Did you eat or drink the tempting food or drink?	(1) 1‐7 Likert scale; 1=not at all intense, 7=extremely intense; (2) yes or no
Craving	How strong is your craving to eat a specific food right now?	Visual analog scale: 1=low intensity to 10=high intensity
Momentary affective experience (or related interoceptive state indicators)	(1) Right now, I feel calm, tense, upset, relaxed, content, worried, hungry, stressed, tired, bored, anxious, overwhelmed, ashamed, energetic; and (2) Right now, I feel very good, good, fairly good, neutral, fairly bad, bad, very bad	(1) 5 Likert scale; 1=not at all, 5=extremely; (2) −5 to 5 Likert scale; −5=very bad, +5=very good

a This will be the question used to calculate the total number of lapses.

b This will be the question used to determine the frequency of endorsed types of lapses, if applicable.

c This question will be used to assess the type of dietary temptations.

d These questions will be used to assess the intensity of and ability to resist dietary temptations.

### Questionnaire Measures

At each assessment, a series of questionnaires will be completed via REDCap. At baseline only, demographic information and food insufficiency will be assessed. Questionnaire measures of self-regulatory mechanisms are found in Table 3 and will be completed at all assessment time points by all participants. At follow-up time points, program satisfaction questionnaires will also be completed, which will be used to assess the acceptability of the IDWL, yoga, and wellness programs. Yoga participants will also be asked to complete several additional questionnaires at follow-up related to their perception of the yoga classes, perceived benefits of practicing yoga [59], and frequency and duration of yoga practice.

**Table 3.** Validated questionnaires administered at baseline, 3-month, 6-month, and 18-month assessments to assess various aspects of self-regulation.

Questionnaire	Assessment	Scoring
Weight Control Strategies [60]	Self-monitoring, exercise, and diet as weight control strategies	0‐90; comprised of a 30-item scale with four component solution for the subscales: (1) dietary choices, (2) self-monitoring strategies, (3) physical activity, and (4) psychological coping
Five Facet Mindfulness Questionnaire [61,62]	Mindfulness (including one’s ability to observe, describe, and act with awareness, and be nonjudgmental and nonreactive to one’s inner experience)	Comprised of 5 subscales: nonjudging (8-40), nonreactivity (7-35), observing (8-40), describing (8-40), and acting with awareness (8-40)
Distress Tolerance Scale [63]	Perceived capacity to tolerate distress	15‐75; higher scores indicate greater distress tolerance
Self-Compassion Scale [64]	Degree to which individuals act compassionate toward themselves when experiencing a difficult time	26‐130; higher scores reflect higher levels of self-compassion
Weight Efficacy Lifestyle Questionnaire [65]	Self-efficacy (one’s confidence in their ability to avoid overeating when faced with certain situations)	20‐100; higher scores indicate greater self-efficacy in managing weight-related behaviors
Behavioral Inhibition System (BIS) and Behavioral Activation System (BAS) [66]	Inhibitory control (measures motivation to avoid aversive outcomes)	BIS: 7‐28; higher BIS scores indicate greater sensitivity to punishment and BAS: 4‐16; higher BAS scores indicate greater sensitivity to reward
Barratt Impulsiveness Scale [67]	Inhibitory control: impulsiveness and includes cognitive, motor, and nonplanning impulsivity	30‐150; higher scores reflect higher impulsivity

### Aerobic PA

Minutes of aerobic PA will be objectively measured for 10 days, at each assessment period, using the previously validated Actigraph accelerometer [68]. Participants will be mailed an accelerometer, provided with instructions on how to wear the device, and be instructed to wear the Actigraph on their waist during all waking hours, except for water-related activities. They will also be asked to keep a log to record when they put the device on, took it off, and any exercise that they may have performed while not wearing the device. When processing the data, only “valid” days (ie, ≥8 hours of Actigraph wear time) will be considered in the analysis. Minutes per day spent in total and bout-related (≥10-minute bouts) moderate-to-vigorous intensity PA will be calculated using validated methods. One common critique of yoga as a WL intervention is that yoga participation may reduce aerobic PA. By objectively assessing PA, this study will be able to empirically answer this question. Upon study completion, at the time of analysis for this study, we will use all recommended processing methods for all accelerometry data and report findings accordingly.

### Adherence Measures

Intervention adherence will include the percentage of IDWL lessons viewed and frequency of self-monitoring energy intake, weight, and aerobic exercise minutes entered into the study website (or transmitted to the study website via the Fitbit app) by participants over the study duration. Attendance at live yoga and wellness classes will also be assessed. Additional adherence metrics in the yoga condition include the number of times each prerecorded yoga video was viewed and weekly minutes per week of yoga practice (reported on the study website). During months 13‐18, self-reported yoga practice will be assessed via monthly surveys.

### Trial Monitoring, Safety, and Adverse Events

Upon study enrollment, participants will be instructed to contact the study staff should they experience any illnesses, injury, or side effects that are distressing to them throughout the 18-month research study. Moreover, each month, randomized participants will be required to complete a brief survey, which asks about any injuries or illnesses over the past month. Should a participant report an injury or illness on this survey, they will be contacted by the research staff to gather more information. Finally, at each assessment period (3, 6, 12, and 18 months), participants will be asked to report any illnesses, injuries, changes in health status, and medications experienced since the previous assessment. All adverse events (AEs; any untoward medical occurrence in a person that is temporarily associated with participation in the clinical study) will be reported by the principal investigator (JU) to the institutional review board, the trial’s data safety monitoring board, and funding sponsor. Serious AEs (any AE that results in 1 or more of the following: death, life-threatening event, inpatient hospitalization, persistent or significant disability or incapacity, and important medical event based on appropriate medical judgment) will be reported within 24 hours. All other AEs will be reported twice yearly and will be included within regular reporting to the data safety monitoring board (consisting of external reviewers who have been approved by the study sponsor).

### Data Analytic Plan and Sample Size Considerations

#### Sample Size Considerations

This study is fully powered for the primary aim, which is to examine treatment effects on WL from baseline through follow-up, taking into account the initial 3-month WL period. Power was calculated using a combination of G*Power and MPlus Monte Carlo estimation. Effect sizes were based on a preliminary study assessing the effects of yoga for WL that showed within the yoga condition, there was significant WL from 3 to 6 months (Cohen *d*=0.45). Effect sizes from both yoga and contact-matched control arms were used to estimate a between-group effect at follow-up to use in the power calculations (difference in median percent WL of 3.5% between conditions among those with high initial WL; 95% CI 0.005-0.139). Assuming a similar effect size, 20% correlation between covariates, a total sample size as low as 146 would yield sufficient power (>80%) for testing between-group effects on WL from baseline. However, the final sample size was inflated to 210 to account for (1) the possibility that the effect size estimates that were obtained from our pilot work and used to determine the sample size were not generalizable, (2) adequately powered secondary aims (secondary outcomes and mediation), and (3) attrition rates similar to what we have seen in past studies by this research team (<20% of randomized sample at the end of treatment).

#### Analysis and Statistical Methods

Prior to analyses, data will be cleaned and explored, including outlier analysis and censoring of implausible values. Sociodemographics (eg, age, biological sex, and race or ethnicity), baseline weight, height, and BMI will be summarized across the aggregate sample and compared between groups using 2-tailed *t* tests for continuous variables, chi-square analyses for categorical variables, and nonparametric tests as appropriate. The distribution of each of the primary and secondary study outcomes will be assessed using both parametric and graphical methods and transformed as necessary (eg, log transform toward normality). Potential distributions for the outcome variables include normal and zero-inflated distributions. *P* values will be adjusted for multiple comparisons using the Hommel method. All analyses will be on the intent-to-treat sample (everyone randomized at 3 months will be included in the final analysis). Sensitivity analyses will explore effects of those who provide information during the postenrollment or prerandomization period that might influence outcomes (eg, initiation of WL medication).

#### Primary Aim

We will examine treatment effects on weight change from baseline to 18 months using a longitudinal mixed effects regression model. Absolute weight will be regressed on treatment assigned (YOGA vs WELLNESS), initial WL (from baseline to 3 months), and identified confounders. Model estimates and group comparisons will be generated for the 12-month primary end point, with contrasts specified to allow for understanding group differences in the postrandomization period (eg, 3‐12 months controlling for baseline to 3 months). Models will include a participant-specific intercept and random slope terms to adjust for repeated measurements within participants over time. Further, we will test for clustering by cohort and adjust, if needed. Mixed models are flexible, in that they allow for a wide range of specifications of the time effect (linear and nonlinear), which is particularly important in WL data, where outcomes do not necessarily follow a linear pattern over time.

#### Secondary Aims

Using a similar analytic approach as that described for the primary aim, we will examine intervention effects on secondary outcomes, including frequency of dietary lapses and potential lapse triggers (eg, affect, cravings, and ability to resist dietary temptations), as assessed via EMA. Namely, longitudinal mixed effects models will be specified that regress outcomes over time on intervention group (YOGA vs WELLNESS), time, intervention*time, baseline value of the outcome, and confounders. Models will also adjust for the clustering of data within participants. Effects of day and measurement period will be tested, and adjustments made, as needed.

To evaluate the secondary aims of this study, we will examine potential mediators (eg, affect, mindfulness, self-compassion, and distress tolerance) of the treatment effect on primary (ie, weight change) and secondary outcomes (ie, dietary lapses) using a multiple mediator model with bootstrapped SEs (10,000 bootstrapped samples). Models estimate the path coefficients (a path: effects of YOGA vs WELLNESS on changes from baseline in potential mediators and b path: effects of changes from baseline in potential mediators on WL or lapse outcomes) as well as the indirect effect of intervention (ab path: YOGA vs WELLNESS on outcomes through the mediators).

#### Exploratory Aims

To examine longer-term treatment effects on weight change, we will generate model estimates and perform group comparisons for the 18-month end point using the mixed effects model of weight described earlier (aim 1). We will also examine moderators of the treatment effect on weight change from baseline to 12-month follow-up, including initial WL (weight change from baseline to 3 months), baseline BMI, race or ethnicity, and biological sex, using a similar analytic approach to that described in the primary aim.

#### Missing Data

Analyses will be on the intent-to-treat sample (everyone randomized will be included in the final analysis) under various assumptions about the missing data mechanism. Sensitivity to these assumptions will be tested. Specifically, we will gather follow-up information and reasons for dropout regardless of protocol completion and censor at the point of loss. We will compare the robustness of our findings using 3 statistical approaches for handling missing data. First, we will use a multiple imputation approach to impute missing outcomes. Next, we will use inverse probability weighting with propensity scores. This is a two-step method: (1) using logistic regression, the probability of missingness is modeled as a function of baseline covariates and baseline values of the outcome and (2) the inverse of the propensity scores (predicted probabilities of dropout from the first step) serves as weights in our regression model of the outcomes. Provided the data are missing at random or that the probability of missingness can be fully explained by observable data, this approach produces asymptotically unbiased estimates. To allow for the possibility that the missing at random assumption may not hold, we will also use a third approach, pattern mixture models, in which the distribution of the outcome is assumed to follow a mixture of 2 distributions: one for those who complete follow-up and another for those who do not.

## Results

This RCT was funded in July 2023 with an original project timeline of July 2023-June 2028. Enrollment began in February 2024 and is ongoing with the expectation of closing enrollment in June 2026. As of the submission of this protocol manuscript, recruitment timelines have been achieved, and 4 of 5 cohorts have launched. Upon study completion, results will be analyzed, and it is expected that the primary outcome findings will be submitted for publication within 6 months of the project end date.

## Discussion

### Principal Findings

This paper provides a summary of the rationale and methods of the PATH Trial. The primary goals of this randomized trial are to examine whether the addition of yoga to behavioral WL treatment improves long-term WL and to elucidate mechanisms through which yoga impacts weight in WL-seeking adults. These findings will have clinical relevance, as overweight and obesity are global health challenges linked to cardiometabolic risks, and despite effective WL interventions, long-term WL remains difficult [35,36]. Therefore, it is crucial to identify and test novel strategies, such as yoga, which have the potential to support sustained WL.

This study results from a synthesis of literature from a variety of disciplines using yoga interventions (cardiometabolic, pain, and addiction fields) and proposes a unique application of yoga to the area of weight management. While these other disciplines tend to focus on the psychological benefits of yoga for improving health, the weight control field has traditionally viewed yoga as a “mode” of exercise and has failed to consider that yoga is a mind-body intervention that could indirectly impact weight via psychological mechanisms (eg, improvements in self-regulation). Therefore, it is important to delineate that a yoga practice is not an exercise modality that produces effects similar to aerobic exercise, which has a higher caloric expenditure and is commonly recommended in WL programs, and therefore will be unlikely to elicit a comparable exercise-induced energy deficit [69]. Unlike past literature, this trial is uniquely designed and fully powered to evaluate self-regulatory pathways through which yoga impacts dietary lapses and WL, representing the first RCT to examine these mechanistic pathways within the context of WL treatment.

This design was chosen as the best method of answering the questions central to the study. For example, the use of an equal contact-time wellness program for the control arm, delivered through the same live, synchronous videoconferencing modality as yoga, provides a stringent test of the relative efficacy of yoga as a complementary therapy to IDWL. The decision to implement yoga at the start of week 14, rather than week 1, was made so that participants can dedicate their focus to initiating important WL skills and achieving program goals at the start of the program (eg, regular self-weighing, self-monitoring, calorie restriction, and aerobic exercise). Adding yoga at week 1 would further increase participant burden, which is already high at this time. Delaying yoga until week 14 allows additional time for these important WL skills to become more routine, which then allows individuals more time to dedicate to practicing yoga. Further, given that maximal WL in IDWL programs is achieved around 3 months [7,52] (earlier than what is observed with in-person treatment [35]), one way to bolster WL at this time would be to add an intervention component, which can both add novelty and strengthen self-regulation. Research supports this approach, as individuals report that over time, boredom increases; it requires more effort to stay on track, and the cost-benefit ratio for WL increases (ie, the effort required to adhere to WL practices is greater than the positive feedback received for adherence) [35,70-74]. This makes continued diligence to self-regulatory WL behaviors challenging, particularly during times of high stress, emotional difficulties, or when exposed to highly palatable foods. Our pilot study, which also implemented yoga following a period of WL treatment, provided further support for this decision [51].

This study is novel, in that it is one of a handful of studies to combine yoga with a WL program, and it is the first to empirically examine this combination of approaches using fully remote intervention procedures (ie, IDWL combined with live, online yoga classes). We made the decision to combine yoga with IDWL treatment from months 4 to 12 (vs stopping IDWL treatment) for the following reasons. First, most WL programs are 1 year in duration, and there is no added cost to continuing the IDWL program, given that it is fully automated. Additionally, as prior research suggests that yoga reinforces self-regulatory skills taught within IDWL, extending the IDWL treatment would provide an opportunity for participants to “practice” these skills. Finally, this approach allows for examination of the combined effect of yoga and IDWL.

Another novel aspect of this study is the use of technology-based assessment methods (EMA) for capturing participants’ dietary lapses and potential lapse triggers (eg, cravings, temptations, and affect) in near-real-time, within a naturalistic setting, while participating in a WL and yoga intervention. We also pair these survey assessment methods with objective monitoring of aerobic PA, so that they occur simultaneously. While pilot work indicates that yoga participation does not reduce aerobic exercise engagement [4], this is a frequent concern regarding the inclusion of yoga into a WL program. Thus, this study will be able to empirically answer whether including yoga reduces engagement in other modalities of exercise while also allowing for novel exploratory analyses related to both the acute and chronic effects of yoga on psychological and dietary variables.

This study will also add to the literature in its ability to examine for whom yoga is most efficacious for producing WL. Examination of yoga within the context of a weight management program is in its infancy, and studies assessing the effect of yoga on body weight have been primarily in the normal weight, non-Hispanic, White female population. This study seeks to enroll ≥35% male individuals and ≥40% individuals from racial and ethnic groups that are commonly underrepresented in WL trials. Thus, we will be in a unique position to explore whether the effect of yoga on WL differs by biological sex and race or ethnicity or whether there are other significant moderators (eg, BMI, 3-month WL, and socioeconomic status) of the effects of yoga on WL. To our knowledge, this is the first study to examine whether there are specific subgroups of WL-seeking individuals who benefit most from yoga participation.

### Conclusions

The proposed study is significant because it is one of the few trials to examine the combined effect of yoga and WL treatment among individuals with overweight or obesity and the first fully powered trial to assess the combination of yoga and WL beyond 6 months. While the yoga field is often criticized for a lack of scientific rigor, this study represents a significant step toward improving the quality of research in this area, as it is empirically guided and thoughtfully designed to ensure rigor and reproducibility of study findings (eg, randomized trial with contact-matched control, manualized protocols, and fidelity checks). This study also uses and tests an easily disseminated intervention model, which combines a fully automated, low-cost IDWL program with live, online yoga classes; thus, translation potential is high. Examination of mediators and moderators of the effect of yoga on WL is also important for improving theoretical models of yoga and self-regulation, optimizing future intervention trials, and enhancing our understanding of how and for whom yoga impacts long-term WL. Ultimately, if primary hypotheses are confirmed, yoga could be considered a valuable intervention component for improving IDWL treatment. In the future, if favorable results are observed, longer-term follow-up would also be warranted to better understand the role of yoga in WL maintenance over periods of time longer than this study is planning. This study has great potential to make a significant contribution to both yoga and WL fields and addresses critical gaps in the current literature.

## Supplementary material

10.2196/78973 Checklist 1 SPIRIT checklist.

## Abbreviations

AE: adverse event
EMA: ecological momentary assessment
HIPAA: Health Insurance Portability and Accountability Act
IDWL: internet-delivered weight loss
PA: physical activity
PATH: Pathway to Achieving Total Health
RCT: randomized controlled trial
REDCap: Research Electronic Data Capture
SPIRIT: Standard Protocol Items: Recommendations for Interventional Trials
TiDieR: Template for Intervention Description and Replication
WL: weight loss
